# Supplementing young cattle with a rumen-protected grape extract around vaccination increases humoral response and antioxidant defenses

**DOI:** 10.1016/j.vas.2022.100232

**Published:** 2022-01-14

**Authors:** Paul Engler, Clémence Desguerets, Mohamed El Amine Benarbia, Yassine Mallem

**Affiliations:** a Labcom FeedInTech, 42 rue Georges Morel, Beaucouzé 49070, France; b Nor-Feed SAS, 3 rue Amedeo Avogadro, Beaucouzé 49070, France; c Oniris, Nutrition, PathoPhysiology and Pharmacology (NP3) Unit, National College of Veterinary Medicine, Food Science and Engineering, 101 route de Gachet, Nantes 44300, France

**Keywords:** Vaccination, Bovine respiratory disease, Antioxidant, Grape extract, Polyphenols, Antibody

## Abstract

Vaccination is an important mainstay of biosecurity and disease prevention in livestock farming. Vaccination failures represent an economic burden for the farmer. Polyphenol supplementation, known for its antioxidant properties, could help reduce oxidative damage and improve the success of vaccination. We evaluated the effect of a rumen-protected grape extract (RPGE) supplementation around vaccination on the immune response in young ruminants.

22 young female cattle (aged 6 to 8 months), born in the same dairy farm, were randomly divided into 2 groups. One group (BP-O, *n* = 11) was supplemented with a RPGE (Nor-Grape® BP-O, Nor-Feed, France), whilst a control group (CTL, *n* = 11) was not. All animals were vaccinated (D14) with an inactivated vaccine against PI-3 V and BRSV. A booster was given 3 weeks later (D35). Supplementation began 15 days before vaccination (D0) and ended 15 days after the last injection (D49). Antibody titers and total antioxidant status (TAS) were performed on blood samples drawn on D0, D35 and D56.

Results show that the BP-O group tended to have a greater overall antibody response to BRSV and PI-3 V on D56 (*P* < 0.10) and PI-3 V titer was significantly higher in the BP-O group on D35 (*p* < 0.05). A greater total antioxidant capacity (*P*<0.05 at D56) was also observed in the supplemented group. Results also showed a strong correlation between PI-3 V antibody titers and TAS (*p* < 0.001).

Thus, since supplemented animals became seropositive faster and long-term immunity appeared to be improved, this supplementation strategy could be of interest to enhance the immune response during a vaccination episode by reducing oxidative stress.

## 1 Introduction

Over the years, vaccination has become a key element of animal production, in order to maintain the animals’ health and limit the economic impacts of different infectious agents on the herd (Roth, 2011). Bovine Respiratory Disease (BRD) is, for instance, considered to be the costliest infectious agent for bovine production, estimated to cost around $800 to $900 million every year for the American feedlot industry alone (American Society of Animal Science, 2020) or $23.60 per treated calf (Grissett, White & Larson, 2015). BRD can be caused by a variety of pathogens (Grissett et al., 2015) and different vaccinal strategies have been developed to help protect young cattle against it, such as multivalent vaccines (Chamorro & Palomares, 2020).

However, vaccination success is limited by several factors, both exogenous (i.e., good manufacturing, transporting, storing and manipulating practices) and endogenous (Richeson, Hughes, Broadway & Carroll, 2019; Roth, 1999). It has indeed been shown that stress can exert an adverse impact on the immune response and sometimes lead to partial immunosuppression (Amadori & Zanotti, 2016; Inbaraj, Sejian, Bagath & Bhatta, 2016). This phenomenon could involve oxidative disbalance resulting from excessive free radical production associated with poor antioxidant defenses, triggering uncontrolled inflammation which, in turn, impairs the immune function (Amadori & Zanotti, 2016; Lauridsen, 2019). Oxidative stress could thus potentially counteract the correct immunization of the vaccinated animal.

To counteract oxidative stress, several antioxidant compounds are often used. Antioxidants for which minimal requirements have been established (vitamin E, vitamin C, selenium) but also natural compounds with an antioxidant property, more recently discovered, such as polyphenols are also of interest. Their strong antioxidant capacity and their synergistic effect with other antioxidants has indeed been shown in multiple studies (Brenes, Viveros, Chamorro & Arija, 2016; Gessner, Ringseis & Eder, 2017; Iglesias, Pazos, Torres & Medina, 2012). Extensive studies reported the intricate relationship between polyphenols’ antioxidant properties, anti-inflammatory effect and their link with immunomodulation in different animal species (Ding, Jiang & Fang, 2018; Mir & Agrewala, 2008; Shakoor et al., 2021). Studies have also evidenced the potential interest of grape polyphenols to stimulate humoral immunity in birds (Ao & Kim, 2020; Farahat, Abdallah, Ali & Hernandez-Santana, 2017) and in rabbits (Hassan, Mahrose & Basyony, 2016). Recent work also showed a general effect of grape polyphenols on dairy cows’ immunity from a transcriptomic point of view (Pauletto et al., 2020). However, to the best of our knowledge, no published research has been carried out on the effect of grape polyphenols supplementation in cattle on their adaptive immunity. Furthermore, studies have shown that grape polyphenols’ antioxidant activity is degraded in ruminal fluid (Chedea et al., 2016), thus requiring a potential protection to preserve their activity. Therefore, the aim of this work was to evaluate the effect of a supplementation in young cattle on their humoral response, with a low dose of rumen-protected grape extract around a vaccination period.

## 2 Material and methods

### 2.1 Reagents

Metmyoglobin and ABTS (2, 2′-amino-di-[3-*ethylbenzthiazoline sulphonate*]) present in the chromogen reagent, peroxide hydrogen (substrate), standard and control reagents were purchased from the Randox laboratory (Randox Lab., Ltd., UK).

### 2.2 Animal recruitment

All procedures were performed according to ethical standards for the care and use of animals and with respect of good handling practices for cattle. Animal welfare through the study was respected and supervised by the senior livestock specialist recruited for this study. The study took place in a commercial organic farm in North-Western France during the late winter period. Informed written consent was obtained from the farm manager prior to the research being carried out. 22 young Prim'Holstein heifers were recruited. Since maternal antibodies have been shown to persist in the calf blood for up to 6 months (Chamorro et al., 2014), the animals were selected to be 6–8 months old at the beginning of the trial in order to be free of maternal immunity interference. All animals were born and raised on the farm and thus shared the same environmental, feed and herd management conditions. They were all checked by a veterinarian and only healthy animals, having never been vaccinated against the pathogen agents targeted by the vaccines used in the study, were recruited for this study. In order to avoid bias, any health issue and/or medicated treatment administered during the period of the study was considered a reason for exclusion of the animal from the study.

Cattle body weight was estimated for all animals at the beginning of the experiment using a barymetric ribbon and measuring, the thoracic perimeter at the withers, just behind the anterior legs. The mean estimated weight at the beginning of the experiment was 161 ± 17 kg and both groups did not differ significantly.

Vaccine injections and blood samplings were carried out by a trained veterinarian.

### 2.3 Diets

The basal diet of the animals was constituted as follows: ad libitum straw and 4 kg per day of a mixture of 49% corn grain, 27.5% rapeseed cake, 7% wheat straw, 7% hay, 7% molasses, 2% minerals and 0.5% salt. Detailed composition of the commercial mineral used (Vital Concept, France) is presented in Table 1.

**Table 1:** Analytical composition of the mineral used and daily uptake per animal.

Analytical constituent	Per kg of mineral	Per animal/day (80 g)
Phosphorus	5%	4 g
Calcium	25%	20 g
Magnesium	5%	4 g
Zinc (as zinc oxide)	7,000 mg	560 mg
Manganese (as manganese oxide)	5,000 mg	400 mg
Copper (as copper sulfate)	1,500 mg	120 mg
Iodine (as anhydrous calcium iodine)	120 mg	9.6 mg
Cobalt (as tetrahydrate cobalt (II) acetate)	40 mg	3.2 mg
Selenium (as sodium selenite + as microencapsulated sodium selenite)	25 mg (15 mg + 10 mg)	2.0 mg (1.2 mg + 0.8 mg)
Vitamin A	500,000IU	40,000IU
Vitamin D3	120,000IU	9,600IU
Vitamin E	1,500IU	120IU

The 22 heifers were randomly divided between two groups: a control group (CTL, *n* = 11) and a supplemented group (BP-O, *n* = 11). Both groups received the same basal diet and the animals from the BP-O group were fed an additional supplementation of 670 mg of a commercial rumen-protected grape extract (RPGE) per animal and per day (Nor-Grape® BP-O, Nor-Feed, France) for 7 weeks. The inclusion rate of the RPGE corresponded to the manufacturer's recommendations.

To ensure good uptake of the supplementation, it was homogeneously diluted by the manufacturer in of a mixture of calcium carbonate and crushed wheat, which was distributed on top of the diet using a pre-measured spoon. The CTL group received a pre-measured spoon of the blank dilution matrix to avoid bias. The farmer distributed daily 10 g of the diluted supplementation or the blank dilution matrix per animal per day and checked their uptake by the heifers. A particular appetence for the matrix (blank or supplemented) was reported by the farmer with total consumption observed daily.

The RPGE contained >60% total polyphenols, mainly constituted of oligomeric and monomeric flavanols and anthocyanins (suppliers data sheet), rumen-protected through a micro-encapsulation with an organic plant-based matrix. Polyphenol percent was measured by using the spectrophotometric method of total polyphenol quantification at 280 nm (European Commission, 2017) and confirmed this value.

### 2.4 Vaccination protocol

Vaccination was carried out according to the manufacturer recommendations, by subcutaneous injection of 2 mL of the vaccine solution in the caudal fold. Heifers received a first injection of a combination of inactivated Para Influenza 3 Virus (PI3V) and inactivated Bovine Respiratory Syncytial Virus (BRSV, BOVALTO RESPI 3®, Merial, France) on D15 of the supplementation protocol. A second booster dose of both vaccines was then administered on D35 of the supplementation period.

### 2.5 Blood sampling and analysis

Blood samples were taken from the heifers’ coccygeal vein on D1, D35 and D56. Samples for antibody titrations were drawn on dry tubes, then refrigerated and sent to an independent laboratory for direct analysis. Samples for total antioxidant status were drawn on heparin tubes and frozen for later analysis. All analyses were carried out blindly.

#### 2.5.1 Specific antibody titration

Specific antibody titration was carried out for both vaccines on D1, D35 and D56 samples by serum neutralization. Briefly, two-fold dilutions of serum (from pure to 1:2048) were mixed with 25 µl of the working virus before being incubated for 1 h at 37 °C. 150 µl of a cell suspension of MDBK (Madin Darby Bovine Kidney) cells were then added in each well. In the absence of antibodies in the sera, the non-neutralized viruses would infect the MDBK cells. Wells showing cytopathogenic effects were scored after 3 days of incubation.

#### 2.5.2 Total antioxidant status

Total antioxidant status (TAS) assay was carried out on D1, D35 and D56 samples to establish the TAS at the beginning and the end of the experiment. TAS was measured by total antioxidant quantification using ABTS^+^ (2,2′-Azino-di-[3-*ethylbenzthiazoline sulphonate*]) radical formation with the commercially available Randox Total Antioxidant Status test kit (Randox Lab., Ltd., UK). The principle of the assay consists in the fact that the metmyoglobin present in the chromogen reacts with the peroxide hydrogen to form ferrylmyoglobin, a free radical species. The chromogen also contains ABTS which reacts with ferrylmyoglobin to produce the ABTS•+, a radical cation which has blue-green color and can be measured at 600 nm. Antioxidants present in the added blood sample cause suppression of this color production proportional to their concentration. The analytical procedure indicated by the kit manufacturer was followed. Briefly, two solutions R2 and R3 were prepared using solution R1 (buffer) from the TAS kit. Solution R2 (chromogen) was prepared from a 10 mL vial of chromogen mixed with 10 mL of R1. Solution R3 (substrate) was prepared from 1 mL of substrate mixed with 1.5 mL of R1. Then, wells were filled with 125 µl of R2 (ABTS and metmyoglobin) and 2,5 µl blood sample. Control, standard and reagent blank wells are made on each plate. After shaking and incubation to reach 37 °C, the initial absorbance A₁ at 600 nm was read by a spectrophotometric analyzer. Then, 25 µl of R3 (hydrogen peroxide) was added to each well. The absorbance A₂ was read at 600 nm after shaking and waiting for 3 min. The calculation was made with the following formula: mmol eq Trolox/L = Factor x (ΔA reagent blank-ΔA sample) where, Factor = standard concentration/(ΔA reagent blank-ΔA standard) and A₂ - A₁ = ΔA of sample or standard or reagent blank. Results were then expressed in mmol equivalent Trolox /L.

### 2.6 Statistical treatment

Statistical analyses were carried out using R studio (version 1.4.1106) with multcomp and nlme packages.

The data of neutralizing antibody titer and total antioxidant status were analyzed using a linear mixed effects model. This model for studying the effect of time and groups on the observed value was as follows: Observed values_ij_ ∼ µ + (Time)*_i_* + (Group)*_j_* + (Time * Group) + ε_ij_; where : µ=average; Time = fixed effect of time (*i* = D1, D35 or D56); Group= fixed effect of group (*j*= BP-O or CTL); Time*Group = interaction between Time and Group; ε_ij_ = the residuals. When the interaction of time and group did not significantly affect the studied criterion, a multiple comparison test was performed with Tukey's test, allowing for a pairwise comparison of means (Bretz, Hothorn & Westfall, 2016).

## 3 Results

### 3.1 Humoral response

**Fig. 1:**
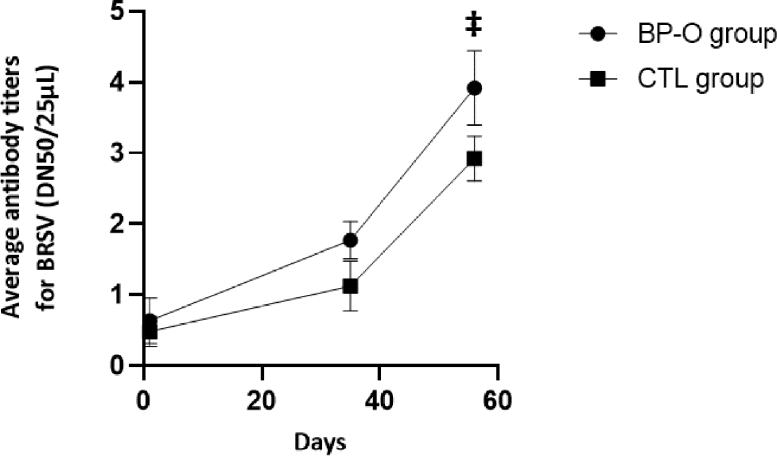
Comparison of BRSV neutralizing antibody titers between groups after vaccination. ‡ 0.05≤*P* ≤ 0.10.

No difference of BRSV-specific antibody titers was observed at the beginning of the study between groups (Fig. 1). Furthermore, both groups presented a very low average antibody titer (0.5 ± 0.2 and 0.6 ± 0.3 for CTL and BP-O group, respectively) signifying an absence of exposure to the BRSV virus before the beginning of the experiment. On D35, 3 weeks after the first injection, no difference was observed between groups for their BRSV-specific circulating antibody titers (1.2 ± 0.3 and 1.8 ± 0.3 for CTL and BP-O group, respectively). However, whilst no statistical differences were observed between antibody titers on D1 and D35 in the CTL group (*p* > 0.05), D35 antibody titers were significantly higher than D1 ones in the BP-O group (*p* < 0.01), signifying a significant seropositivity from the first injection. On D56, 3 weeks after the second injection, the average BRSV-specific antibody titer from BP-O heifers tended to be higher than that from the CTL group (3.9 ± 0.5 vs. 2.9 ± 0.3, respectively, *P* < 0.10). In both groups, antibody titers were significantly higher on D56 than on D35 or D1 (*p* < 0.001).

**Fig. 2:**
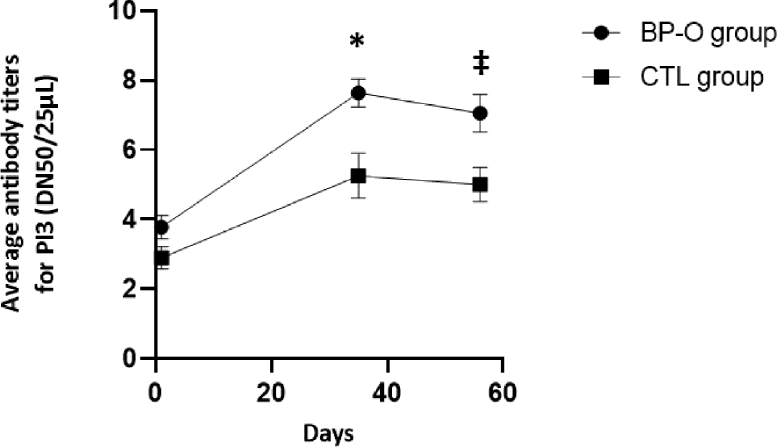
Comparison of PI3 neutralizing antibody titers between groups after vaccination. * *P* < 0.05; ‡ 0.05≤*P* ≤ 0.10.

No difference of PI3V-specific antibody titers was observed at the beginning of the study between groups (Fig. 2). However, both groups presented a medium average antibody titer (2.9 ± 0.3 and 3.8 ± 0.3 for CTL and BP-O group, respectively) signifying the existence of a previous exposure to the PI3V virus before the beginning of the experiment. On D35, 3 weeks after the first injection, the average PI3V-specific antibody titer from BP-O heifers was significantly higher than that from the CTL group (7.6 ± 0.4 vs. 5.3 ± 0.6, respectively, *P* < 0.05). Furthermore, on D56, 3 weeks after the second injection, the average PI3V-specific antibody titer from BP-O heifers also tended to be higher than that from the CTL group (7.1 ± 0.5 vs. 5.1 ± 0.5, respectively, *P* < 0.10). Additionally, on D35 and D56, the antibody titer of both groups was significantly higher than the D1 one (*p* < 0.05).

### 3.2 Total antioxidant status

**Fig. 3:**
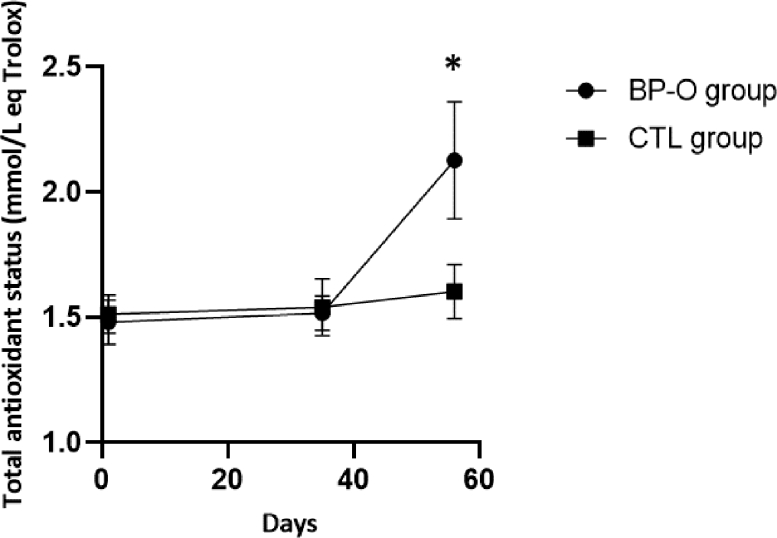
Comparison of total antioxidant status between groups after vaccination. * *P* < 0.05.

No difference was observed between groups for their TAS at the beginning of the experiment (Fig. 3). However, on D56, the average TAS from BP-O heifers was significantly higher than in CTL animals (2.13 ± 0.23 mmol eq. Trolox/L vs. 1.60 ± 0.11 mmol eq. Trolox/L, respectively, *p* < 0.05). Also, whilst BP-O TAS was significantly increased by D56 (*p* < 0.05), no difference between D0 and D56 TAS could be observed in the CTL group.

Furthermore, TAS of animals was strongly correlated with the BRSV-antibody titers of heifers (*R* = 0.4285, *p* < 0.001, Table 2), but not with PI3V-antibody titers.

**Table 2:** Correlation matrix.

Pearson's coefficient	TAS	PI3V titer	BRSV titer
TAS	–	0.1373	0.4285***
PI3V titer		–	0.5226***
BRSV titer			–

*** indicate significant correlation p-value (*p* < 0.001, pairwise two-sided *p*-values).

## 4 Discussion

### 4.1 Humoral response

With higher titers for both BRSV and PI3V in the BP-O group, the supplementation of heifers with 0.67 g of a RPGE/head/day showed a significant impact on the vaccines-specific antibody titers. Interestingly, BRSV-vaccinated animals with high antibody titers following vaccination have been shown to be associated with the absence of clinical signs and of PCR-detectable shed virus after exposure to a viral challenge (Hägglund et al., 2011), evidencing their important part in the herd immunity. Thus, the present findings indicate that the individuals’ antibody titers increase induced by this RPGE supplementation could participate in the herd protection.

Furthermore, interestingly, in the case of cattle encountering BRSV for the first time (first vaccine injection), the vaccination alone did not induce significant changes in specific antibody titers in the CTL group (*p* > 0,05, D35), whilst the supplemented heifers did show a significantly higher titer (*p* < 0.01), underlining a positive impact of the supplementation from the first injection. Thus, evidencing a beneficial effect of the supplementation on the humoral response of the studied cattle.

The increase in specific circulating antibody titers observed in heifers supplemented with a RPGE is in accordance with other research where the effect of a long-time supplementation with grape pomace in dairy cattle (inclusion level: 5% of the total daily ration) was studied (Pauletto et al., 2020). The authors reported that the supplementation had a positive impact on immunity-related gene expression. However, the effect on the humoral immunity was not measured. Whilst, to the best of our knowledge, this other work is the sole reported study of grape polyphenols’ effect on ruminants’ immunity, several studies have been carried out on the effect of grape polyphenols on monogastric immune response. Interestingly, the low inclusion dose used in the present study, corresponding to 400 mg of total polyphenols/head/day, still induced a positive effect on the humoral response, despite being much smaller than the 23.4 g total polyphenol intake/day from a supplementation with grape pomace observed in this reported other work (19.5 kg DMI with 7.5% GP at 1.6% total polyphenols, (Pauletto et al., 2020).

Increased circulating antibody titers following a supplementation with a grape extract in combination with vitamin E (Iqbal et al., 2015) or with 7.5% grape pomace (Ebrahimzadeh, Navidshad, Farhoomand & Aghjehgheshlagh, 2018) have indeed been reported in broiler chickens. Higher circulating immunoglobulin G (IgG) levels, complement 4 (C4) and interleukin 2 (IL-2) were also found in ducks supplemented with 100 ppm and 200 ppm of grape extract (Ao & Kim, 2020) evidencing a profound implication of grape polyphenols in the immune system response. Similar findings were also shared in swine, where a supplementation with 100 ppm and 150 ppm of grape seed extract in weaning piglets resulted in higher circulating levels of IgG, IgM, C4 and IL-2 (Hao et al., 2015), confirming findings observed in ducks (Ao & Kim, 2020). Higher circulating levels of IgG and IgM in sows were also reported following a supplementation with 200 ppm or 300 ppm of grape extract (Wang et al., 2019). Interestingly, in swine, only grape extract-based supplementations showed a beneficial effect on the immune system, since the use of other sources of grape polyphenols such as grape seed meal did not result in an increase of immunoglobulin levels (A, M or G) in piglets (Pistol, Palade, Marin, Stancu & Taranu, 2019).

Collectively, all these findings support the view that grape extract polyphenols could play a role in the modulation of the adaptive immunity of supplemented animals. Furthermore, whilst extensive research has been carried out on grape polyphenols following the discovery of the “French Parad'Ox”, several reviews have confirmed the role of polyphenols in the immunity of Multiple species (Ding et al., 2018; Mir & Agrewala, 2008; Shakoor et al., 2021). Findings indicate that the action of polyphenols is not only that of an antioxidant role, but they also play a part in the downregulation of inflammation pathways and stimulate the immune system and defenses production (i.e., immunoglobulins) (Gessner et al., 2017; Shakoor et al., 2021). The present results consolidate this hypothesis since heifers with a higher antioxidant level showed a higher humoral response. A strong correlation observed between the TAS of heifers and their BRSV-antibody titers indeed further reinforced this hypothesis.

### 4.2 Total antioxidant status

The increase in total antioxidant status observed in heifers supplemented with a rumen-protected grape extract are in accordance with the extensive literature on the antioxidant effect of grape polyphenols in different species (Brenes et al., 2016; Gessner et al., 2017).

More specifically, the present results are in line with observations made by other authors. In one study where reported grape seed extract inclusion levels ranged from 20 to 80 mg of grape extract per kg of body weight in dairy cows averaging 580 kg liveweight (thus equivalent to 11.6 g to 46.4 g/head/day), supplemented cows had a significant improvement of their total antioxidant capacity in the post-partum period (up to 42 days post-partum) (Huang et al., 2019). Moreover, a supplementation with 10 g of grape extract/head/day was reported to be able to control calving-induced oxidative stress through modulation of superoxide dismutase (SOD) expression in peri‑parturient cows (Colitti & Stefanon, 2006). In sheep, a single acute dose of grape extract (10% of the dry matter intake), injected directly in the rumen, was reported to confer a significant increase of the total antioxidant status in sheep for up to 30 h after administration through the production of derived phenolic metabolites (Gladine, Rock, Morand, Bauchart & Durand, 2007).

Interestingly, the inclusion level used in the present study (0.67 g/head/day) was significantly lower than that used in the discussed literature but still allowed to induce a significant effect, suggesting that the rumen-protection was able to protect the polyphenols’ activity from degradation by the ruminal fluid evidenced described elsewhere (Chedea et al., 2016) and the level of grape polyphenols was able to increase TAS in supplemented cattle. Furthermore, whilst a wide diversity of phenolic compounds exists within grape extracts, the low molecular weight of the polyphenols from the present extract (oligomeric and monomeric flavanols, anthocyanins) are known to be small enough to be absorbed at the intestinal level, where bigger polymeric condensed tannins are incapable of being absorbed (Brenes et al., 2016). Thus, good bioavailability of the phenolic compounds found in the present grape extract, associated with a rumen-protection, could explain why beneficial effects could be observed despite a very small dose.

## 5 Conclusion

Consequently, the findings of the present study are compliant with the observations reported on monogastric species, namely, the improvement of antioxidant defenses in supplemented animals leading to the increase humoral response. They underline the interest of a such a supplementation in young cattle around the vaccination events in order to promote a good antioxidant protection and humoral response and, in turn, a successful immunization associated with a better immune protection due to higher circulating antibody levels.

Further research is needed to better characterize the effect of a supplementation with a rumen-protected grape extract on the immune system of ruminants and the biological pathways involved that result in the stimulation of the humoral response.

## Funding

This research work was constitutive of C.D.’s veterinary thesis. C.D. received funding from ONIRIS for this work and complementary financial support from Nor-Feed to cover the costs of the blood analyses.

## Ethics statement

All authors have been personally and actively involved in the manuscript and are jointly and individually responsible for their content.

## CRediT authorship contribution statement

**Paul Engler:** Writing – original draft, Writing – review & editing. **Clémence Desguerets:** Visualization, Conceptualization, Visualization, Data curation, Formal analysis, Writing – original draft, Writing – review & editing. **Mohamed El Amine Benarbia:** Writing – original draft, Writing – review & editing. **Yassine Mallem:** Visualization, Data curation, Formal analysis, Writing – original draft, Writing – review & editing.

## Declaration of Competing Interest

The authors declare that they have no known competing financial interests or personal relationships that could have appeared to influence the work reported in this paper. The authors declare the following financial interests/personal relationships which may be considered as potential competing interests: Paul Engler reports a relationship with Nor-Feed that includes: employment. Mohammed El Amine BENARBIA reports a relationship with Nor-Feed that includes: employment.

## References

[bib0001] Amadori M., Zanotti C. (2016). Immunoprophylaxis in intensive farming systems : The way forward. Veterinary Immunology and Immunopathology.

[bib0002] American Society of Animal Science (2020). Preview : Economic effects of bovine respiratory disease. Journal of Animal Science.

[bib0003] Ao X., Kim I.H. (2020). Effects of grape seed extract on performance, immunity, antioxidant capacity, and meat quality in Pekin ducks. Poultry Science.

[bib0004] Brenes A., Viveros A., Chamorro S., Arija I. (2016). Use of polyphenol-rich grape by-products in monogastric nutrition. A review. Animal Feed Science and Technology.

[bib0005] Bretz F., Hothorn T., Westfall P. (2016).

[bib0006] Chamorro M.F., Palomares R.A. (2020). Bovine respiratory disease vaccination against viral pathogens. The veterinary clinics of North America. Food Animal Practice.

[bib0007] Chamorro M.F., Walz P.H., Haines D.M., Passler T., Earleywine T., Palomares R.A. (2014). Comparison of levels and duration of detection of antibodies to bovine viral diarrhea virus 1, bovine viral diarrhea virus 2, bovine respiratory syncytial virus, bovine herpesvirus 1, and bovine parainfluenza virus 3 in calves fed maternal colostrum or a colostrum-replacement product. Canadian Journal of Veterinary Research.

[bib0008] Chedea V.S., Pelmus R.S., Cismileanu A.E., Pistol G.C., Palade L.M., Taranu I. (2016). Total polyphenols content, antioxidant activity and stability of a grape pomace incorporated in animal feed. Scientific Papers Animal Science and Biotechnologies.

[bib0009] Colitti M., Stefanon B. (2006). Effect of natural antioxidants on superoxide dismutase and glutathione peroxidase mRNA expression in leukocytes from periparturient dairy cows. Veterinary Research Communications.

[bib0010] Ding S., Jiang H., Fang J. (2018). Regulation of immune function by polyphenols. Journal of Immunology Research.

[bib0011] Ebrahimzadeh S.K., Navidshad B., Farhoomand P., Aghjehgheshlagh F.M. (2018). Effects of grape pomace and vitamin E on performance, antioxidant status, immune response, gut morphology and histopathological responses in broiler chickens. South African Journal of Animal Science.

[bib0012] European Commission (2017). Commission implementing regulation (EU) 2017/307-Of 21 February 2017-concerning the authorisation of dry grape extract of vitis vinifera spp. Vinifera as a feed additive for all animal species except for dogs. OJ.

[bib0013] Farahat M.H., Abdallah F.M., Ali H.A., Hernandez-Santana A. (2017). Effect of dietary supplementation of grape seed extract on the growth performance, lipid profile, antioxidant status and immune response of broiler chickens. Animal : an international journal of animal bioscience.

[bib0014] Gessner D.K., Ringseis R., Eder K. (2017). Potential of plant polyphenols to combat oxidative stress and inflammatory processes in farm animals. Journal of Animal Physiology and Animal Nutrition.

[bib0015] Gladine C., Rock E., Morand C., Bauchart D., Durand D. (2007). Bioavailability and antioxidant capacity of plant extracts rich in polyphenols, given as a single acute dose, in sheep made highly susceptible to lipoperoxidation. British Journal of Nutrition.

[bib0016] Grissett G.P., White B.J., Larson R.L. (2015). Structured literature review of responses of cattle to viral and bacterial pathogens causing bovine respiratory disease complex. Journal of Veterinary Internal Medicine.

[bib0017] Hägglund S., Hu K., Vargmar K., Poré L., Olofson A.S., Blodörn K. (2011). Bovine respiratory syncytial virus ISCOMs-Immunity, protection and safety in young conventional calves. Vaccine.

[bib0018] Hao R., Li Q., Zhao J., Li H., Wang W., Gao J. (2015). Effects of grape seed procyanidins on growth performance, immune function and antioxidant capacity in weaned piglets. Livestock Science.

[bib0019] Hassan F.A., Mahrose K.M., Basyony M.M. (2016). Effects of grape seed extract as a natural antioxidant on growth performance, carcass characteristics and antioxidant status of rabbits during heat stress. Archives of Animal Nutrition.

[bib0020] Huang Y., Oikonomou G., Hu J., Li Y., Du X., Du Y. (2019). Effect of feeding grape seed Proanthocyanidin extract on production performance, metabolic and anti-oxidative status of dairy cattle. Arquivo Brasileiro de Medicina Veterinária e Zootecnia.

[bib0021] Iglesias J., Pazos M., Torres J.L., Medina I. (2012). Antioxidant mechanism of grape procyanidins in muscle tissues : Redox interactions with endogenous ascorbic acid and α-tocopherol. Food Chemistry.

[bib0022] Inbaraj S., Sejian V., Bagath M., Bhatta R. (2016). Impact of Heat Stress on Immune Responses of Livestock: A Review. *Pertanika Journal of Tropical Agricultural Science*.

[bib0023] Iqbal Z., Kamran Z., Sultan J., Ali A., Ahmad D.S., Shahzad M.imran (2015). Replacement effect of vitamin E with grape polyphenols on antioxidant status, immune and organs histopathological responses in broilers from one to thirty-five days of age. The Journal of Applied Poultry Research.

[bib0024] Lauridsen C. (2019). From oxidative stress to inflammation : Redox balance and immune system. Poultry Science.

[bib0025] M. A. Mir., J. N. Agrewala (2008). "Dietary Polyphenols in Modulation of the Immune System". In Neville Vassallo (ed.). Polyphenols and Health: New and Recent Advances. Nova Publishers. pp. 245. ISBN 978-1-60456-349-8.

[bib0026] Pauletto M., Elgendy R., Ianni A., Marone E., Giantin M., Grotta L. (2020). Nutrigenomic effects of long-term grape pomace supplementation in dairy cows. Animals.

[bib0027] Pistol G.C., Palade L.M., Marin D.E., Stancu M., Taranu I. (2019). The effect of grape wastes, wine industry byproducts, on inflammatory and antioxidant biomarkers in post-weaning piglets. Lucrări Științifice - Universitatea de Științe Agricole Şi Medicină Veterinară, Seria Zootehnie.

[bib0028] Richeson J.T., Hughes H.D., Broadway P.R., Carroll J.A. (2019). Vaccination management of beef cattle. The veterinary clinics of North America. Food Animal Practice.

[bib0029] Roth J.A. (1999). Mechanistic bases for adverse vaccine reactions and vaccine failures. Advances in Veterinary Medicine.

[bib0030] Roth J.A. (2011). Veterinary vaccines and their importance to animal health and public health. Procedia in Vaccinology.

[bib0031] Shakoor H., Feehan J., Apostolopoulos V., Platat C., Al Dhaheri A.S., Ali H.I. (2021). Immunomodulatory effects of dietary polyphenols. Nutrients.

[bib0032] Wang X., Jiang G., Kebreab E., Yu Q., Li J., Zhang X. (2019). Effects of dietary grape seed polyphenols supplementation during late gestation and lactation on antioxidant status in serum and immunoglobulin content in colostrum of multiparous sows1. Journal of Animal Science.

